# The effects of exercise session timing on weight loss and components of energy balance: midwest exercise trial 2

**DOI:** 10.1038/s41366-019-0409-x

**Published:** 2019-07-09

**Authors:** Erik A. Willis, Seth A. Creasy, Jeffery J. Honas, Edward L. Melanson, Joseph E. Donnelly

**Affiliations:** 1 Center for Health Promotion and Disease Prevention, University of North Carolina-Chapel Hill, Chapel Hill, NC USA; 2 Division of Epidemiology and Genetics, Metabolic Epidemiology Branch, National Cancer Institute, Bethesda, MD USA; 3 Division of Endocrinology, Metabolism, and Diabetes, Anschutz Medical Campus, University of Colorado Aurora, Aurora, CO USA; 4 Department of Internal Medicine, The University of Kansas Medical Center, Kansas City, KS USA; 5 Division of Geriatric Medicine, Department of Medicine, University of Colorado Anschutz Medical Campus, Aurora, CO USA; 6 Eastern Colorado VA Geriatric Research, Education, and Clinical Center, Denver, CO USA

**Keywords:** Risk factors, Metabolism

## Abstract

**Background/objectives:**

Circadian physiology has been linked to body weight regulation and obesity. To date, few studies have assessed the association between exercise timing and weight related outcomes. The aim of this secondary analysis was to explore the impact of exercise timing (i.e., 24 h clock time of exercise session) on weight loss and components of energy balance.

**Subjects/methods:**

Overweight/obese (BMI 25.0–39.9 kg/m^2^), physically inactive, young adults (~51% female) completed a 10-month supervised exercise program (400 or 600 kcal/session for 5 days/week) or served as non-exercise controls (CON). Participants were categorized based on the time of day in which they completed exercise sessions (Early-Ex: >50% of sessions completed between 7:00 and 11:59 am; (*n* = 21), Late-Ex: >50% of sessions completed between 3:00 and 7:00 pm; (*n* = 25), Sporadic-Ex: <50% of sessions completed in any time category; (*n* = 24), and CON; (*n* = 18)). Body weight, energy intake (EI; digital photography), and non-exercise physical activity (NEPA; accelerometer) were assessed at baseline, 3.5, 7, and 10 months. Total daily energy expenditure (TDEE; doubly labeled water), was assessed at baseline and 10 months.

**Results:**

At month 10, weight loss was significantly greater in both Early-EX (−7.2 ± 1.2%; *p* < 0.001) and Sporadic-EX (− 5.5 ± 1.2%; *p* = 0.01) vs CON (+0.5 ± 1.0%), and Early-EX vs Late-EX (−2.1 ± 1.0%; *p* < 0.001). There were no between group differences for change in TDEE, EI, and non-exercise energy expenditure (*P* > 0.05). A significant group × time interaction (*p* = 0.02) was observed for NEPA (counts/min), however, after adjusting for multiple comparisons, group effects were no longer significant.

**Conclusions:**

Despite minimal differences in components of energy balance, Early-EX lost significantly more weight compared with Late-Ex. Although the mechanisms are unclear, the timing of exercise may be important for body weight regulation.

## Introduction

High volumes of exercise are associated with weight loss and the prevention of weight gain [1]. In fact, studies utilizing supervised exercise, to confirm the completion of the recommended dose of exercise, have found that engaging in >250 min of exercise per week elicits clinically significant weight loss (>5%) [2–5]. However, there are large inter-individual differences in weight loss despite participants engaging in similar amounts of exercise [6–11]. It has been postulated that these differences in weight loss are owing to biological (e.g., reductions in resting metabolic rate (RMR) and total daily energy expenditure (TDEE)) and behavioral (e.g., changes in non-exercise physical activity (NEPA) or energy intake (EI)) compensation, resulting in less weight loss than theoretically predicted by the amount of energy expended from exercise [12–14]. However, the literature regarding biological or behavioral changes in response to exercise is inconclusive and conflicting [15–19]. More recently, evidence has suggested that the timing of exercise may play a critical role in body weight regulation [20–22]. However, the contribution of timing of exercise, within the 24 h day, to exercise induced weight loss is unclear.

Circadian physiology has been linked to body weight regulation and obesity [23]. The circadian system has been shown to play an important role in regulating daily rhythms of metabolism, sleep/wake cycle, feeding behavior, and hormonal secretions [24]. Most evidence for the role of circadian rhythms and body weight regulation is related to the timing of EI [25–29] and sleep/wake cycle [21, 23, 30–32]. To date, the few studies that have assessed the association between exercise timing and weight related outcomes have been limited by study design and the lack of objective assessment methods [20–22]. Owing to the high levels of variability in exercise induced weight loss, further understanding the role of exercise timing could be important to maximize weight loss efforts. In this secondary analysis, data from the recently completed Midwest Exercise Trial 2 (MET-2) afforded an opportunity to assess whether timing of exercise had differential effects on changes in weight, EI, RMR, non-exercise energy expenditure (NExEE), NEPA, and sedentary time in men and women in response to a 10-month supervised exercise training program with verified levels of exercise energy expenditure (ExEE).

## Methods

Participants were recruited into MET-2 (Registration Clinical Trial number: NCT01186523, www.clinicaltrials.gov) and provided written informed consent before engaging in any aspect of the trial and were compensated for participation. The approval for this study was obtained from the human subjects committee of the University of Kansas-Lawrence. Briefly, MET-2 was a 10-month randomized efficacy trial, 5 day/week supervised exercise intervention at two levels of ExEE (400 or 600 kcal/session) or non-exercise control that was designed to evaluate the effect of aerobic exercise, without energy restriction, on weight loss in sedentary overweight and obese men and women. Blinding of participants to group assignment was not possible. Investigators and research assistants were blinded at the level of outcome assessments and data entry [16, 33]. A detailed description of the design and methods for MET-2 [33], results for the primary outcome [6], changes in NExEE and NEPA [16], and differences between intervention responders and non-responders have been published [34].

### Participants

Participants were physically inactive (planned physical activity <500 kcal/wk. as assessed by recall [35], overweight/obese, (BMI 25–40 kg/m^2^) young adults (age 18–39 years) who were physically able to exercise. Individuals were ineligible to participate in the study if they had history of chronic disease (i.e., diabetes, heart disease, etc.), elevated blood pressure (>140/90), lipids (cholesterol, >6.72 mM; triglycerides, >5.65 mM), or fasting glucose (>7.8 mM), used tobacco products, were taking medications that would affect physical performance (i.e., beta blockers, metabolism, thyroid, or steroids), or were unable to perform laboratory assessments.

### Exercise training and group classification

Over the 10-month (40 weeks) exercise intervention, participants were asked to complete 200 exercise sessions. Exercise sessions were supervised in a university exercise facility that was open from 7:00 am to 7:00 pm Monday–Friday and 8:00 am to 12:00 pm Saturdays. Exercise sessions consisted of primarily treadmill walking/jogging 5 days/week [33]. Alternate activities (stationary biking, walking/jogging outside) were permitted for one day/week to provide variety and to decrease the risk of overuse injuries. Exercise progressed from 150 kcal/session to the target exercise energy expenditure of 400 or 600 kcal/session (original study groups of the MET-2 trial) at the end of month four and remained at target for the final six months. Weekly exercise logs were maintained by research staff to track exercise session start times, intensity, and duration and were verified by heart rate monitors. Exercise session start times from participant daily exercise logs were used to determine when during the 24-hour day participants completed each session. Proportion of sessions completed during each hour the exercise facility was open were aggregated into early, mid-day, and late sessions over the 10-month intervention. Early sessions were defined based on exercise being completed in the morning hours of 7:00–11:59 am. The remaining facility operation hours were then dichotomized into mid-day (early afternoon) hours of 12:00–2:59 pm and late (late afternoon) hours of 3:00–7:00 pm. Participants were then classified as an early exerciser (Early-Ex; *n* = 21) if they completed ≥50% of their total sessions between the hours of 7:00–11:59 am, mid-day exerciser (*n* = 11) if completing ≥50% of their total sessions between the hours of 12:00–2:59 pm, a late exerciser (Late-Ex; *n* = 25) if completing ≥50% of their total sessions between the hours 3:00–7:00 pm, or a sporadic exerciser (Sporadic-Ex; *n* = 24) if they did not complete ≥50% of their total sessions in any time category. This categorical scheme was based off of previous exercise timing research [36, 37]. Owing to small sample size for the mid-day exercisers this category was removed from further analyses.

### Control group

Participants assigned to the non-exercise control group (CON) were instructed to maintain their typical patterns of physical activity and dietary intake over the duration of the 10-month study.

### Anthropometrics (height, weight, body composition)

Body weight was measured between 7:00–10:00 am following a 12 hour fast while wearing a standard hospital gown using a digital scale accurate to ± 0.1 kg (PS6600, Befour, Saukville, WI). Height was measured using a stadiometer (Model PE‐WM‐60‐84, Perspective Enterprises, Portage, MI) and BMI was calculated as weight (kg)/height (m)^2^. Dual energy X-ray absorptiometry (DXA) was used to determine fat-free mass (FFM), fat mass (FM) and percent body fat (Lunar DPX-IQ). Women completed a pregnancy test prior to each DXA.

### Components of energy expenditure

RMR was assessed at baseline and 10 months by open circuit indirect calorimetry. Participants reported to the laboratory between 6:00 and 10:00 am after a 12 hour fast and 48-hour abstention from aerobic exercise [38] and rested quietly for 15 min in a temperature controlled (21–24 °C) isolated room. Subsequently, participants were placed in a ventilated hood for assessment of VO_2_ and VCO_2_ for a minimum of 35 min using a ParvoMedics TrueOne 2400 indirect calorimetry system (ParvoMedics Inc., Sandy, UT). Criteria for a valid RMR was a minimum of 30 min of measured values with < 10% average standard deviation across the last 30 min of the minimum 35-minute assessment. Absolute RMR (kcal/d) was calculated using the Weir equation [39]. In order to account for the effects of changes in body composition on RMR, we also calculated adjusted RMR and exercise energy expenditure by adding residuals from RMR = fat mass + fat-free mass and exercise energy expenditure = fat mass + fat-free mass, respectively, to the corresponding mean values.

TDEE was assessed by DLW over 14 days at baseline and 10 months. The 10-month assessment was obtained during the final two weeks of the exercise training protocol. Participants reported to the laboratory between 8:00–9:00 am following an overnight fast. Baseline urine specimens were collected prior to oral dosing with a mixed solution of 0.10 g of 99% atom percent excess (APE) ^2^H_2_O and 0.15 g of 10% APE ^18^O per kilogram of body weight. Following oral administration, the dose bottle was rinsed with 100 mL of tap water and consumed by the participant. A weighed 1:400 dilution of each participant’s dose was prepared, and a sample of the tap water was stored at − 70°C for later analysis. Additional urine samples were collected on days 1 and 14. On these days, two urine samples were collected at least 3 hours apart. All urine samples were stored in sealed containers at − 70 °C before analysis. Samples were analyzed in duplicate for ^2^H_2_O and H_2_^18^O by isotope ratio mass spectrometry, as previously described by Herd et al. [40]. TDEE was estimated using the equation of Elia [41], as follows: total energy expenditure (MJ/d) = (15.48/RQ + 5.55) × rCO_2_ (L/d) and then values were converted to kcal/d. ExEE was assessed by treadmill walking/running at baseline and monthly during the intervention using indirect calorimetry (ParvoMedics Inc., Sandy, UT) at 1 min intervals. For the ExEE assessment, participants performed a brief warm-up (~2 min) followed by an exercise session (~15 min) at 70% (±4 beats/minute) and 80% (±4 beats/minute) of heart rate maximum. ExEE (kcal/minute) was calculated as the average ExEE over the 15-minute exercise session. The duration of exercise periods was obtained from exercise logs maintained by research staff and verified by an HR monitor. NExEE, i.e., energy expenditure not associated with exercise training, was calculated as follows: [(TDEE × 0.9)−RMR]−net exercise energy expenditure. This approach assumes that the thermic effect of food represents 10% of TDEE [42]. Note that net exercise energy expenditure at baseline and for CON at both time points equals zero.

Similarly to RMR, TDEE was also calculated relative to body composition (fat mass and fat-free mass). Linear regression was used to calculate adjusted TDEE for each subject by adding residuals from TDEE = fat mass + fat-free mass regression to mean TDEE at both baseline at 10 months. Subsequently, adjusted NExEE was calculated as [(Adjusted TDEE × 0.9)−adjusted RMR]−net adjusted exercise energy expenditure.

### EI

EI was assessed over 7‐day periods (minimum of two meals/day on weekdays and one meal/day on weekends) of ad libitum eating at baseline and at 3.5, 7, and 10 months in a University of Kansas cafeteria. Two digital photographs (90° and 45° angle) were obtained before and after consumption of each meal with the cafeteria trays placed in docking station to standardize the camera angle. Notes were placed on the tray to identify beverages (e.g., diet vs. regular soft drink; skim vs. whole milk) and other food items that would be difficult to identify from the photo. Foods consumed outside the cafeteria (e.g., snacks, non-cafeteria meals) were assessed using multiple‐pass recalls. Types and amounts of food and beverages consumed at the cafeteria and results from the recalls were entered into the Nutrition Data System for Research (NDS‐R Versions 2005, 2006, University of Minnesota, Minneapolis, MN) for the quantification of EI.

To analyze EI distribution during the day, the starting time of each meal was considered the time of intake. The proportions of EI in the three periods of morning (4:00–10:59 am), afternoon (11:00–4:59 am), and evening (5:00pm–3:59 am) were calculated during the active intervention (months 3.5, 7, and 10), as described by de Castro [43]. The morning period was specifically defined to capture most of “breakfast” but not lunch. Similarly, the afternoon period was defined to capture most of “lunch” and afternoon snack, whereas the evening period was determined to capture most of “dinner” and evening snack [43].

### NEPA/sedentary time

NEPA was assessed by an accelerometer (Actigraph GT1M, Pensacola, FL) worn at the waist, over the non-dominant hip, for 7 consecutive days, using 1‐minute epochs with a minimum of 10 hours constituting a valid day. Three valid days were required to be included in the analysis. No minimum criteria for number of weekdays or weekend days were required. Non-wear time was identified as ≥60 consecutive minutes with 0 counts/min, with allowance for 1–2 min of accelerometer counts between 0 and 100 [44]. Data were processed using a custom SAS program. NEPA (≥100 counts/min) was calculated by removing accelerometer data over the duration of exercise sessions from the daily accelerometer data. Sedentary time was defined as time during wear time with accelerometer readings <100 counts/min [44]. Data are reported as proportion of total wear time spent in sedentary, light physical activity (LPA), and moderate-vigorous intensity physical activity (MVPA). On average, approximately six valid days with over 14 hours of wear time of accelerometer data were available. There were no differences in wear time between the groups throughout the intervention.

### Statistical analysis

Baseline demographic and outcome variables were summarized by means and standard deviations. For TDEE, RMR, and NExEE, the main outcome was change over the intervention period, which was calculated as the 10-month value minus the baseline value. Pearson correlation coefficients were used to assess the correlation between proportion of exercise sessions completed early, mid-day, late, and weight change at month 10. To test for differences in average change between the four groups, analysis of covariance was used. General linear mixed models, were used to assess the impact timing of exercise sessions (early, late, sporadic, and non-exercise controls), time (treated as a categorical variable; baseline, 3.5, 7, 10 months), and the group-by-time interaction effects on weight and non-exercise physical activity outcomes.

Several error covariance structures were assessed and Toeplitz covariance was used because the Bayesian Information Criterion was smaller. All analyses were adjusted for age, sex, original randomization group, and corresponding baseline outcome value. To investigate whether the relation of weight change and exercise timing group were modified by sex or ExEE, we performed multiplicative interaction of these variables by adding their cross-product to the statistical models. The raw or model-based group means were pairwise compared using a Bonferroni-correction for inflation in Type I error. Values are presented as adjusted means and standard error unless otherwise stated. For all models, assumptions of constant variance and normality of the residuals were assessed by visual inspection of residual plots. Statistical significance was determined at 0.05 alpha level and all analyses were performed using.

## Results

### Participants

The baseline characteristics of the 88 participants included in this analysis are shown in Table 1. The sample mean age was ~23 years, BMI was ~31 kg/m^2^, and was composed of ~51% women. Because of technical problems or failure to comply with the assessment protocols, this report includes DLW data from 87 at baseline (Early-EX, *n* = 21; Late-EX, *n* = 25; Sporadic-EX *n* = 23; CON, *n* = 18) and 79 participants at 10 months (Early-EX, *n* = 16; Late-EX, *n* = 24; Sporadic-EX, *n* = 23; CON, *n* = 17) as well as accelerometer data from 88 participants at baseline (Early-EX, *n* = 21; Late-EX, *n* = 25; Sporadic-EX, *n* = 24; CON, *n* = 18) and 84 participants at 10 months (Early-EX, *n* = 20; Late-EX, *n* = 25; Sporadic-EX *n* = 24; CON, *n* = 15). There were no differences in baseline characteristics or weight loss between those that completed all tests and those with missing data. Those in the Sporadic-Ex group were significantly younger (21.2 ± 2.3 years) compared with Early-Ex (23.7 ± 3.5 years; *p* = 0.032) and Late-Ex (24.2 ± 3.1 years; *p* = 0.003). There was no significant difference in ExEE between Early-EX (528.3 ± 105.0 kcal/session), Late-EX (490.3 ± 102.7), or Sporadic-EX (493.7 ± 98.9; *p* = 0.362).

**Table 1:** Sample characteristics

	Early-EX (*n* = 21)		Late-EX (*n* = 25)		Sporadic-EX (*n* = 24)		Controls (*n* = 18)		*p* value
	Mean	SD	Mean	SD	Mean	SD	Mean	SD	
Age (yrs.)^a,b^	23.7	3.5	24.2	3.1	21.2	2.3	22.6	3.0	0.003
Weight (kg)	88.1	16.5	96.9	19.7	87.8	17.5	87.4	14.6	0.173
BMI (kg/m^2^)	29.7	3.6	32.0	5.5	30.6	4.9	29.5	3.6	0.246
Body composition (kg)									
Fat mass	33.5	8.4	36.6	9.5	34.3	11.2	34.1	7.7	0.658
Fat-free mass	51.1	10.1	55.9	13	48.9	9.6	49.2	9.7	0.086
Time of exercise sessions (%)									
Early sessions (7:00 am–11:59 am)^c,d,e^	69.6	14.1	19.9	11.7	40.2	7.5	-	-	<0.0001
Mid-day sessions (12:00 pm–2:59 pm)^d,e^	11.4	10.5	12.8	9.8	27.0	10.6	-	-	<0.0001
Late sessions (3:00 pm or later)^c,d,e^	17.6	13.0	65.9	11.9	31.3	11.2	-	-	<0.0001
	**N**	**%**	**N**	**%**	**N**	**%**	**N**	**%**	
Female (*n* %)	10	47.6	11	44.0	15	62.5	50	9.0	0.601
Randomized group (*n* %)									0.424
400 kcal/session	8	38.1	14	56.0	13	54.2	-	-	
600 kcal/session	13	61.9	11	44.0	11	45.8	-	-	

Data are presented as mean and standard deviation (SD) unless otherwise stated. yrs. = years, cm = centimeters, kg = kilogram, m = meters, Ex = exerciser

^a^Significant differences Sporadic-EX vs. Early-EX *p* < 0.05

^b^Significant differences Sporadic-EX vs. Early-EX *p* < 0.01

^c^Significant differences Sporadic-EX vs. Early-EX *p* < 0.0001

^d^Significant differences Sporadic-EX vs. Late-EX *p* < 0.0001

^e^Significant differences Early-EX vs. Late-EX *p* < 0.0001

### Weight change

**Fig. 1:**
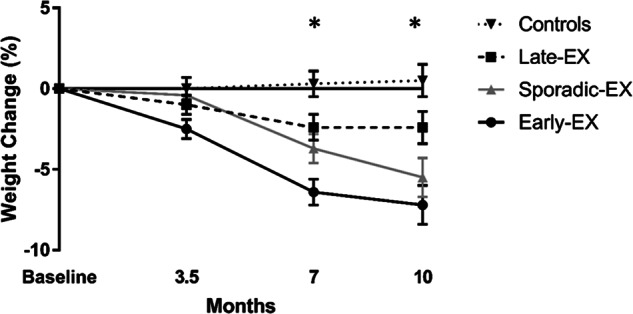
Mixed model results for weight change (%) at months 0, 3.5, 7, and 10 by group. Group effect (*p* = 0.025), time effect (*p* < 0.0001), group × time effect (*p* < 0.0001). *Significant at Month 7: Early-EX > controls (*p* = 0.005), Early-EX > Late-EX (*p* = 0.010). *Significant at month 10: Early-EX > controls (*p* < 0.001), Early-EX > Late-EX (*p* < 0.001), Sporadic-EX > controls (*p* = 0.012)

**Fig. 2:**
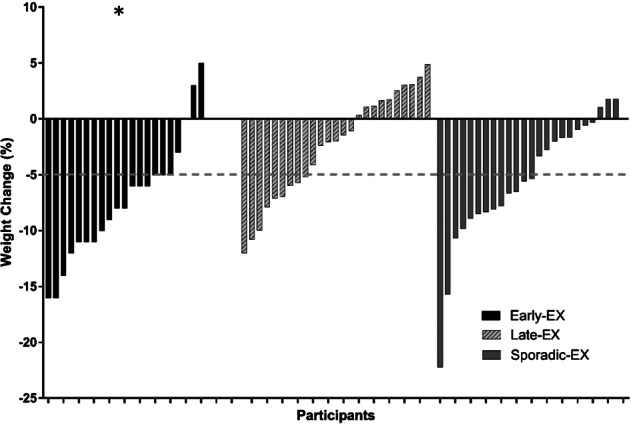
Individual percent body weight change after the 10-month intervention by group. * Significant Early-EX > Late-EX (*p* = 0.007)

As shown in Fig. 1, weight decreased in Early-EX, Late-EX, and Sporadic-EX and was essentially unchanged or slightly increased in the CON. There was a significant group (*p* < 0.025), time (*p* < 0.0001) and group×time interaction (*p* < 0.0001). At month 10, significantly greater weight loss was observed in the Early-EX compared with CON (*p* < 0.001) and Late-EX (*p* < 0.001). In addition, Sporadic-EX had significantly greater weight loss compared with CON (*p* = 0.012). Tests for interaction indicated no statistically significant difference between strata of sex (*p*_interaction_ = 0.304; Figure S1) or original randomization group (*p*_interaction_ = 0.349; Figure S2). There was large variability observed between and within groups regarding weight change over the 10-month intervention (Fig. 2). A significantly greater proportion of individuals in Early-EX (81%) reached clinically meaningful weight loss (−5%) compared with Late-EX (36%; *p* = 0.007). The proportion of Sporadic-EX reaching clinically meaningful weight loss (54%) did not significantly differ between Late-EX (*p* = 0.604) or Early-EX (*p* = 0.172). When all participants were considered together, weight change at month 10 was positively correlated with proportion of exercise sessions completed in the late period (*r* = +0.31; *p* = 0.01; Figure S3), negatively correlated with the proportion of exercise sessions completed in the early period (*r* = −0.39; *p* < 0.001; Fig. S3), but not significantly correlated with proportion of exercise sessions completed in the mid-day.

### Body composition

There were no significant between group differences for change in FFM (*p* = 0.140). FFM was stable from baseline to 10 months in Controls (1.8 ± 0.6 kg), Early-EX (0.1 ± 0.4 kg), Late-EX (0.5 ± 0.4 kg), and Sporadic-EX (−0.01 ± 0.4 kg) groups. FM decreased in Controls (−1.5 ± 1.6 kg), Early-EX (−6.2 ± 1.1 kg), Late-EX (−1.6 ± 0.9 kg), and Sporadic-EX (−3.9 ± 1.6 kg) exercise groups. After adjusting for multiple comparisons, decrease in FM was significantly greater in Early-EX compared with the Late-EX (*p* = 0.005) group. No other significant between groups differences for changes in FM were observed (all *p* > 0.05).

### Change in TDEE, RMR, and NExEE

Mean 10-month changes from baseline in TDEE, RMR, and NExEE for the four groups are shown in Table 2. TDEE increased in Early-EX (+297 ± 377 kcal/day), Late-EX (226 ± 574 kcal/day), and Sporadic-EX (195 ± 606 kcal/day) and decreased in CON (−12 ± 726 kcal/d), however there were no significant group differences for change in TDEE. RMR was relatively unchanged in CON (+24 ± 237 kcal/day), Late-EX (+45 ± 189), Early-EX (−56 ± 237 kcal/day), and Sporadic-EX (−79 ± 236 kcal/day) groups. There were no significant between or within-group differences for change in RMR. Similarly, NExEE was relatively unchanged in CON (−34 ± 719 kcal/day), Late-EX (−105 ± 510 kcal/day), Early-EX (+28 ± 446 kcal/day), and Sporadic-EX (+12.1 ± 545 kcal/day) groups with no significant between or within-group differences for change.

**Table 2:** A summary of the ANCOVA results for unadjusted and adjusted NExEE, TDEE, and RMR presented by group

	Baseline				10 Months				Change				*p* value
	*N*	Mean	95% CI		*N*	Mean	95% CI		*N*	Mean	95% CI		
Fat-free mass (kg)													
Controls	18	49.2	44.3	54.0	18	50.4	44.8	55.9	18	1.8	0.6	3.0	0.140
Early-EX	21	51.1	46.5	55.7	21	51.6	46.9	56.3	21	0.1	−0.7	0.9	
Late-EX	25	55.9	50.6	61.3	25	56.6	51.6	61.6	25	0.5	−0.3	1.2	
Sporadic-EX	24	48.9	44.8	52.9	24	48.7	44.9	52.6	24	0.0	−0.7	0.7	
Fat mass (kg)^a^													0.008
Controls	18	34.1	30.3	37.9	18	34.3	29.7	38.8	18	−1.5	−4.7	1.8	
Early-EX	21	33.5	29.6	37.3	21	26.6	23.2	30.0	21	−6.2	−8.3	−4.1	
Late-EX	25	36.6	32.7	40.5	25	34.4	30.0	38.8	25	−1.6	−3.5	0.2	
Sporadic-EX	24	34.3	29.5	39.0	24	30.4	25.3	35.4	24	−3.9	−5.8	−1.9	
TDEE (kcal/d)													0.462
Controls	18	2725	2296	3154	17	2736	2356	3117	17	−12	−385	362	
Early-EX	21	2637	2367	2907	16	2830	2551	3109	16	297	97	498	
Late-EX	25	3207	2903	3511	24	3466	3217	3714	24	226	−16	468	
Sporadic-EX	23	2713	2439	2988	23	2884	2609	3158	22	195	−74	463	
TDEE Adj. for FM and FFM (kcal/day)													0.469
Controls	18	2851	2577	3125	17	2765	2537	2993	17	−96	-450	259	
Early-EX	21	2693	2523	2864	16	2960	2766	3153	16	333	124	542	
Late-EX	25	3014	2761	3266	24	3260	3024	3495	24	242	15	469	
Sporadic-EX	23	2850	2661	3039	23	3043	2809	3277	22	212	-55	478	
RMR (kcal/d)													0.073
Controls	18	1634	1492	1776	17	1651	1484	1819	17	24	−98	146	
Early-EX	21	1759	1619	1899	16	1675	1485	1866	16	−56	−182	70	
Late-EX	25	1825	1658	1992	24	1875	1715	2036	24	45	−35	125	
Sporadic-EX	23	1681	1530	1832	23	1607	1494	1720	22	−79	−184	25	
RMR Adj. for FM and FFM (kcal/day)													0.673
Controls	18	1706	1626	1786	17	1680	1568	1792	17	−11	−140	118	
Early-EX	21	1794	1711	1876	16	1745	1635	1856	16	−41	−173	91	
Late-EX	25	1714	1646	1781	24	1749	1678	1819	24	45	−28	118	
Sporadic-EX	23	1758	1684	1832	23	1707	1637	1777	22	−62	−150	27	
NExEE (kcal/d)													0.470
Controls	18	818	516	1120	17	811	534	1088	17	−34	−404	335	
Early-EX	21	614	454	775	16	576	390	762	16	28	−210	266	
Late-EX	25	1061	844	1279	24	980	767	1192	24	−105	−321	110	
Sporadic-EX	23	761	612	910	23	742	533	951	22	12	−230	254	
NExEE Adj. for FM and FFM (kcal/day)													0.568
Controls	18	860	598	1122	17	809	564	1054	17	−75	−439	289	
Early-EX	21	630	491	770	16	623	435	811	16	46	−188	279	
Late-EX	25	999	772	1225	24	921	691	1150	24	−91	−299	116	
Sporadic-EX	23	807	666	947	23	786	583	988	22	10	−228	248	

*Adj.* adjusted, *FM* fat mass, *FFM* fat-free mass, *TDEE* total daily energy expenditure, *RMR* resting metabolic rate, *NExEE* non-exercise energy expenditure, *EX* exerciser

^a^Significant differences early-EX vs. late-EX *p* < 0.001

### NEPA and sedentary time

**Fig. 3:**
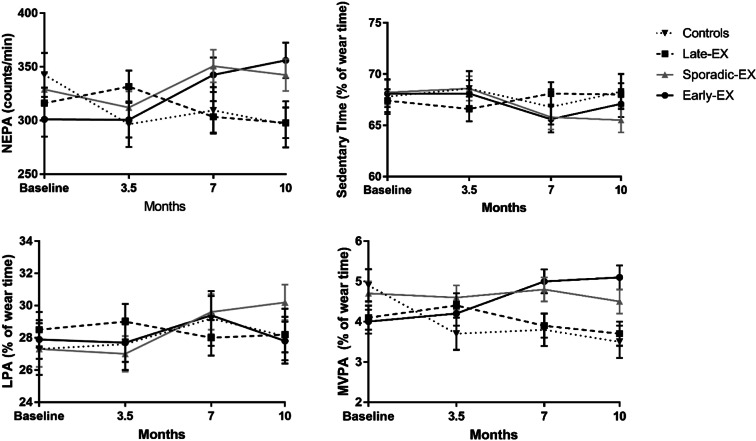
Mixed model results mean accelerometer activity counts per minute (counts/min) and proportion of time spent in sedentary, light (LPA), and moderate-vigorous activity (MVPA) over the 10 months by group. NEPA (counts/min): group × time effect (*p* = 0.028). MVPA (% of wear time): group effect (*p* = 0.040), group * time effect (*p* = 0.03)

As shown in Fig. 3, NEPA (average counts/min) slightly decreased in CON and was essentially unchanged or slightly increased in the exercise intervention groups. There was a significant group × time interaction (*p* = 0.023), however, there were no significant group or time effects. Figure 3 also presents the time spent in sedentary, LPA and MVPA assessed by accelerometer, expressed as a percentage of wear time (excluding exercise). There were no significant effects for group, time, or group × time interaction for sedentary time. For LPA, there were no significant between-or within-group differences (group or time effects) or group × time interactions. For MVPA, there was no significant time effect. Significant group effect (*p* = 0.04) and group × time interaction (*p* = 0.03) was observed. After adjusting for multiple comparisons, the group effects were no longer significantly different.

### EI

Energy and macronutrient intake over the 10-month intervention is presented in Table 3. Mixed modeling results revealed that after controlling for age, sex, original randomization group, and corresponding baseline EI value there were no significant between-or within-group differences (group or time effects) or group × time interactions in absolute (kcal/day) total energy, protein, carbohydrate, or fat intake. In addition, there were no significant group or time effects for proportion of calories consumed from protein or carbohydrates. Relative EI significantly increased over the 10 months in Early-EX, Late-EX, Sporadic-EX and CON (time effect *p* = 0.003). Proportion of calories consumed of fat significantly decreased over the 10 months in Early-EX, Late-EX, Sporadic-EX, and CON (time effect *p* = 0.012). There were no significant effects for group or group×time interaction for relative EI or proportion of calories from fat intake.

**Table 3:** A summary of adjusted means and 95% confidence intervals (CI) from the mixed model results for energy and macronutrient intake presented by group across the 10-month intervention

	Early-Ex			Late-Ex			Sporadic-Ex			Controls		
	Mean	95% CI		Mean	95% CI		Mean	95% CI		Mean	95% CI	
Energy intake (kcal/day)												
Baseline	2817	2615	3020	2864	2683	3046	2846	2659	3033	2988	2715	3260
3.5 months	2677	2474	2880	2848	2667	3030	2658	2470	2845	2841	2568	3114
7 months	2801	2598	3004	2878	2697	3060	2756	2569	2943	2753	2481	3026
10 months	2754	2551	2957	2985	2804	3167	2729	2542	2916	2851	2578	3124
Energy intake (kcal/kg/day)^b^												
Baseline	31.8	29.5	34.1	32.0	29.9	34.1	32.1	30.0	34.2	34.3	31.1	37.4
3.5 months	30.9	28.6	33.2	31.4	29.3	33.5	30.2	28.0	32.3	32.0	28.9	35.2
7 months	33.7	31.4	36.0	33.0	30.9	35.1	32.8	30.7	34.9	31.3	28.2	34.4
10 months	33.2	30.9	35.5	33.8	31.8	35.9	33.2	31.0	35.3	32.3	29.2	35.5
Protein (kcal/day)												
Baseline	96.9	90.6	103.2	101.5	95.9	107.1	104.9	99.1	110.7	109.2	100.4	118.1
3.5 months	98.9	92.6	105.2	99.9	94.3	105.6	101.0	95.2	106.8	110.0	101.2	118.9
7 months	100.5	94.2	106.8	101.0	95.4	106.6	102.4	96.6	108.2	106.6	97.8	115.5
10 months	103.3	97.0	109.6	104.5	98.8	110.1	103.3	97.5	109.1	109.9	101.0	118.7
Carbohydrate (kcal/day)												
Baseline	338.8	320.7	357.0	340.7	324.5	356.9	315.4	298.7	332.1	305.7	280.0	331.4
3.5 months	330.1	311.9	348.4	349.7	333.5	365.9	324.7	308.0	341.4	317.0	291.4	342.7
7 months	322.6	304.4	340.7	340.3	324.1	356.5	319.5	302.8	336.2	316.5	290.9	342.2
10 months	332.0	313.8	350.2	336.3	320.0	352.6	323.8	307.1	340.5	319.4	293.7	345.0
Fat (kcal/day)												
Baseline	117.2	110.6	123.8	114.5	108.6	120.4	123.6	117.5	129.8	119.5	110.2	128.7
3.5 months	119.2	112.5	125.8	112.6	106.7	118.6	121.3	115.2	127.4	112.6	103.4	121.9
7 months	121.1	114.4	127.7	114.5	108.6	120.4	125.1	119.0	131.2	115.0	105.7	124.3
10 months	118.6	112.0	125.3	112.6	106.7	118.5	118.9	112.8	125.0	114.3	105.0	123.5
Protein (%)												
Baseline	14.1	13.0	15.1	14.4	13.5	15.3	14.7	13.8	15.7	15.9	14.4	17.3
3.5 months	14.5	13.5	15.5	14.2	13.3	15.2	14.4	13.5	15.4	16.1	14.6	17.5
7 months	14.7	13.6	15.7	14.3	13.4	15.2	14.5	13.6	15.5	15.5	14.1	17.0
10 months	14.8	13.8	15.8	14.4	13.5	15.4	14.6	13.7	15.6	15.9	14.5	17.4
Carbohydrate (%)												
Baseline	48.3	45.6	51.1	48.9	46.5	51.4	45.3	42.7	47.9	43.1	39.2	47.0
3.5 months	48.0	45.2	50.8	49.3	46.8	51.7	46.3	43.8	48.9	45.2	41.3	49.2
7 months	46.2	43.4	48.9	49.2	46.8	51.7	45.9	43.3	48.4	44.8	40.9	48.7
10 months	47.3	44.5	50.1	47.4	44.9	49.9	46.2	43.6	48.7	45.5	41.6	49.4
Fat (%)^a^												
Baseline	37.0	34.7	39.2	36.3	34.3	38.3	39.3	37.2	41.3	38.6	35.4	41.8
3.5 months	37.1	34.8	39.3	36.3	34.3	38.3	38.5	36.5	40.6	35.9	32.7	39.1
7 months	38.4	36.2	40.7	36.0	34.0	38.1	39.5	37.4	41.6	36.7	33.5	39.9
10 months	37.3	35.0	39.6	35.4	33.4	37.4	36.7	34.6	38.8	36.2	33.0	39.4

*kcal* kilocalorie, *kg* kilogram, *EX* Exerciser

^a^Significant time effect *p* < 0.05

^b^Significant time effect *p* < 0.01

The proportion of total daily EI consumed in the morning hours (4:00 am–10:59 am) was significantly lower in CON (17.8 ± 8.5%) compared with the Early-EX (24.0 ± 8.8%; *p* = 0.006) and Sporadic-EX (22.8 ± 6.0%; *p* = 0.027). Proportion of EI consumed in the morning was not significantly different between Early-EX, Late-EX, or Sporadic-EX (*p* > 0.05). There were no significant differences found between groups for afternoon or evening EI (*p* > 0.05).

## Discussion

The aim of this secondary analysis was to assess daily timing of exercise on weight loss in overweight and obese young adults who completed a 10-month moderate-to-vigorous intensity aerobic exercise program, with ad libitum eating. Of those randomized to exercise, approximately one-third of the sample were categorized into each Early-EX, Late-EX, and Sporadic-EX groups. We found when exercise is supervised and prescribed at a sufficient magnitude, individuals who performed more exercise sessions in the morning had significantly greater reductions in weight compared with those who performed more exercise sessions in the evening. Interestingly, there appeared to be a dose response relationship between proportion of exercise sessions completed in the morning and weight change at 10 months. Weight change at month 10 was inversely correlated with the proportion of exercise sessions completed in the morning and positively correlated with the proportion of exercise sessions completed in the late period. Furthermore, a higher proportion of Early-EX reached clinically significant weight loss (>5%) compared with Sporadic-EX and Late-EX. ExEE, verified by indirect calorimetry, was nearly identical in each group and participants were removed from the study if session attendance dropped below 80%, thus eliminating the likelihood that the variability in weight loss was owing to differential compliance with the exercise prescription. In support of our findings, a previous study by Alizadeh et al. [20] also found that women who exercised in the morning lost significantly more weight (1–2 kg) during 6-weeks of supervised aerobic exercise compared with women who exercised later in the day. In that study, the exercise stimulus was 3 days/week of 30 min of treadmill running, which is much lower than our exercise dose, which may explain why the magnitude of weight change was much lower. Thus, although few studies have considered the effects of exercise timing on weight loss, results of these studies suggest that morning exercise is more effective than afternoon or evening exercise at inducing weight loss.

Although not significant, there were trends for changes in the components of energy balance that would promote a larger degree of negative energy balance in Early-Ex that could potentially explain our findings. EI tended to be greater in Late-EX across the 10 months (Table 3). During the intervention, EI for Late-EX was 80–230 kcal/day higher than Early-EX and 200–250 kcal/day higher than Sporadic-EX. Conversely, NEEx and NEPA slightly decreased in Late-EX but slightly increased in Early-EX and Sporadic-EX. Moreover, the increase in TDEE was ~100 kcal/day higher in Early-EX compared with Late-EX. Interestingly, the increase in TDEE was not significantly lower (~30 kcal/day) in Sporadic-EX compared to Late-EX, however, Sporadic-EX achieved higher weight loss. Thus, it is possible that these small differences compounded over time may have contributed to the observed differences in weight change.

Our results regarding the effect of exercise time during the 24-hour day on EI is in general agreement with the results from the limited number of studies that have compared EI between early and late exercisers [20, 45]. For Example, Maraki et al. [45]. compared acute changes in EI, assessed using a 24-hour diet record, in 12 healthy weight young adult females following morning and evening control (1-hour rest) and morning and evening exercise (1-hour class of aerobic and muscle conditioning) sessions. Relative EI on exercise days was significantly lower than on control days, however, neither absolute nor relative EI was affected by the time of exercise. Alizadeh et al. [20], in a 6-week trial, found no between group differences for change in absolute EI assessed by (24-hour diet record) in a sample of women randomized to morning (*n* = 25) or evening (*n* = 23) exercise, although the difference was nearly significant (*p* = 0.06) with morning exercisers reducing EI by ~ 350 kcal/day and evening exercisers reducing EI ~ 30 kcal/day. In the current study, we also examined differences in the timing of EI finding that Early-EX and Sporadic-EX consumed a higher proportion of EI in the morning compared with CON, but no other differences were observed. Thus, it does not appear that the observed differences in weight loss were owing to timing of EI.

Strengths of the parent study bolster the conclusions of the current analysis, including the randomized efficacy intervention study, inclusion of both men and women, supervised exercise at verified levels of ExEE and times during the 24-hour day, and the use of multiple objective measurements of EI, TDEE, RMR, NExEE, NEPA, and sedentary time. However, because this was a secondary analysis, this study was not designed or powered to detect differences between Early and Late exercisers. Thus unobserved confounders may impact these results as participants were not randomized to early or late exercise, instead these exercise times were self-selected and may be influenced by other factors (e.g., school and work schedules). In addition, we did not include assessments of sleep duration or quality, appetite, or eating behaviors, such as cognitive restraint, uncontrolled eating or emotional eating, menstrual cycle stage or contraceptive use in women, all of which may have provided additional insights into differences in weight loss. Finally, the small sample size and multiple group comparisons limited the ability to explore subgroup (e.g., sex-specific) differences.

This study, combined with the results of previous studies, supports the hypothesis that engaging in morning exercise may result in more weight loss compared to engaging in a similar amount of exercise later in the day. Furthermore, we observed individuals who performed most of their exercise sessions in the afternoon or evening tended to have slightly higher levels of EI and reduced NEPA and NEEx, suggesting that there are potentially important differences in the components of energy balance based on time of day exercise is performed. Prospective randomized trials are needed to confirm these findings.

## Supplementary information

Supplemental Figure Legend

Figure S1

Figure S2

Figure S3

## References

[1] Donnelly JE, Blair SN, Jakicic JM, Manore MM, Rankin JW, Smith BK (2009). American College of Sports Medicine Position Stand. Appropriate physical activity intervention strategies for weight loss and prevention of weight regain for adults. Med Sci Sports Exerc.

[2] Ross R, Dagnone D, Jones PJ, Smith H, Paddags A, Hudson R (2000). Reduction in obesity and related comorbid conditions after diet-induced weight loss or exercise-induced weight loss in men: a randomized, controlled trial. Ann Intern Med.

[3] Ross R, Janssen I, Dawson J, Kungl AM, Kuk JL, Wong SL (2004). Exercise‐induced reduction in obesity and insulin resistance in women: a randomized controlled trial. Obes Res.

[4] Donnelly JE, Honas JJ, Smith BK, Mayo MS, Gibson CA, Sullivan DK (2013). Aerobic exercise alone results in clinically significant weight loss for men and women: midwest exercise trial 2. Obesity.

[5] Flack KD, Ufholz K, Johnson L, Fitzgerald JS, Roemmich JN (2018). Energy compensation in response to aerobic exercise training in overweight adults. Am J Physiol Regul Integr Comp Physiol.

[6] Donnelly JE, Honas JJ, Smith BK, Mayo MS, Gibson CA, Sullivan DK (2013). Aerobic exercise alone results in clinically significant weight loss for men and women: midwest exercise trial 2. Obesity.

[7] King NA, Hopkins M, Caudwell P, Stubbs RJ, Blundell JE (2008). Individual variability following 12 weeks of supervised exercise: identification and characterization of compensation for exercise-induced weight loss. Int J Obesity.

[8] Donnelly JE, Hill JO, Jacobsen DJ, Potteiger J, Sullivan DK, Johnson SL (2003). Effects of a 16-month randomized controlled exercise trial on body weight and composition in young, overweight men and women: the Midwest Exercise Trial. Arch Intern Med.

[9] Bouchard C, Tremblay A, Despres JP, Theriault G, Nadeau A, Lupien PJ (1994). The response to exercise with constant energy intake in identical twins. Obes Res.

[10] Caudwell P, Hopkins M, King NA, Stubbs RJ, Blundell JE (2009). Exercise alone is not enough: weight loss also needs a healthy (Mediterranean) diet?. Public Health Nutr.

[11] Hopkins M, Gibbons C, Caudwel P, Hellstrom PM, Naslund E, King NA (2014). The adaptive metabolic response to exercise-induced weight loss influences both energy expenditure and energy intake. Eur J Clin Nutr.

[12] Donnelly JE, Smith BK (2005). Is exercise effective for weight loss with ad libitum diet? Energy balance, compensation, and gender differences. Exerc Sport Sci Rev.

[13] Major GC, Doucet E, Trayhurn P, Astrup A, Tremblay A (2007). Clinical significance of adaptive thermogenesis. Int J Obesity.

[14] Melanson EL, Keadle SK, Donnelly JE, Braun B, King NA (2013). Resistance to exercise-induced weight loss: compensatory behavioral adaptations. Med Sci Sports Exerc.

[15] Herrmann SD, Willis EA, Honas JJ, Lee J, Washburn RA, Donnelly JE (2015). Do changes in energy intake and non-exercise physical activity affect exercise-induced weight loss? Midwest Exercise Trial-2. Obesity (Silver Spring, Md.).

[16] Willis EA, Herrmann SD, Honas JJ, Lee J, Donnelly JE, Washburn RA (2014). Nonexercise energy expenditure and physical activity in the Midwest Exercise Trial 2. Med Sci Sports Exerc.

[17] Donnelly JE, Herrmann SD, Lambourne K, Szabo AN, Honas JJ, Washburn RA (2014). Does increased exercise or physical activity alter ad-libitum daily energy intake or macronutrient composition in healthy adults? A systematic review. PloS ONE.

[18] Washburn R, Lambourne K, Szabo A, Herrmann S, Honas J, Donnelly J (2014). Does increased prescribed exercise alter non‐exercise physical activity/energy expenditure in healthy adults? A systematic review. Clin Obesity.

[19] Shook RP (2016). Obesity and energy balance: what is the role of physical activity?. Expert Rev Endocrinol Metab.

[20] Alizadeh Z, Younespour S, Rajabian Tabesh M, Haghravan S (2017). Comparison between the effect of 6 weeks of morning or evening aerobic exercise on appetite and anthropometric indices: a randomized controlled trial. Clin Obesity.

[21] Anothaisintawee T, Lertrattananon D, Thakkinstian A, Reutrakul S (2018). The relationship among morningness-eveningness, sleep duration, social jet lag and body mass index in Asian patients with prediabetes. Front Endocrinol.

[22] Alizadeh Z, Mostafaee M, Mazaheri R, Younespour S (2015). Acute effect of morning and afternoon aerobic exercise on appetite of overweight women. Asian J Sports Med.

[23] Westerterp-Plantenga MS (2016). Sleep, circadian rhythm and body weight: parallel developments. Proc Nutr Soc.

[24] Huang W, Ramsey KM, Marcheva B, Bass J (2011). Circadian rhythms, sleep, and metabolism. J Clin Invest.

[25] Aljuraiban GS, Chan Q, Oude Griep LM, Brown IJ, Daviglus ML, Stamler J (2015). The impact of eating frequency and time of intake on nutrient quality and Body Mass Index: the INTERMAP Study, a Population-Based Study. J Acad Nutr Diet.

[26] Arble DM, Bass J, Laposky AD, Vitaterna MH, Turek FW (2009). Circadian timing of food intake contributes to weight gain. Obesity (Silver Spring, Md.).

[27] Garaulet M, Gomez-Abellan P, Alburquerque-Bejar JJ, Lee YC, Ordovas JM, Scheer FA (2013). Timing of food intake predicts weight loss effectiveness. Int J Obes (2005).

[28] Wang JB, Patterson RE, Ang A, Emond JA, Shetty N, Arab L (2014). Timing of energy intake during the day is associated with the risk of obesity in adults. J Hum Nutr Diet.

[29] Maukonen Mirkka, Kanerva Noora, Partonen Timo, Männistö Satu (2018). Chronotype and energy intake timing in relation to changes in anthropometrics: a 7-year follow-up study in adults. Chronobiology International.

[30] Malone SK. Does chronotype modify the relationship between sleep duration and body mass index in adolescents? University of Pennsylvania, 2015.

[31] Olds TS, Maher CA, Matricciani L (2011). Sleep duration or bedtime? Exploring the relationship between sleep habits and weight status and activity patterns. Sleep.

[32] Roenneberg T, Allebrandt KV, Merrow M, Vetter C (2012). Social jetlag and obesity. Curr Biol.

[33] Donnelly JE, Washburn RA, Smith BK, Sullivan DK, Gibson C, Honas JJ (2012). A randomized, controlled, supervised, exercise trial in young overweight men and women: the Midwest Exercise Trial II (MET2). Contemp Clin Trials.

[34] Herrmann SD, Willis EA, Honas JJ, Lee J, Washburn RA, Donnelly JEJO (2015). Energy intake, nonexercise physical activity, and weight loss in responders and nonresponders: the midwest exercise trial 2. Obesity (Silver Spring).

[35] Taylor HL, Jacobs DR, Schucker B, Knudsen J, Leon AS, Debacker G (1978). A questionnaire for the assessment of leisure time physical activities. J Chronic Dis.

[36] Bond DS, Raynor HA, Thomas JG, Unick J, Webster J, Ryder B (2017). Greater adherence to recommended morning physical activity is associated with greater total intervention-related physical activity changes in bariatric surgery patients. J Phys Act Health.

[37] Chomistek AK, Shiroma EJ, Lee I-MJJoPA (2016). The relationship between time of day of physical activity and obesity in older women. J Phys Act Health.

[38] Haugen HA, Melanson EL, Tran ZV, Kearney JT, Hill JO (2003). Variability of measured resting metabolic rate. Am J Clin Nutr.

[39] Weir JB (1949). New methods for calculating metabolic rate with special reference to protein metabolism. J Physiol.

[40] Herd SL, Vaughn WH, Goran MI (2000). Comparison of zinc reduction with platinum reduction for analysis of deuterium-enriched water samples for the doubly labeled water technique. Obes Res.

[41] Elia M. Converting carbon dioxide production to energy expenditure. *The Doubly-Labelled Water Method for Measuring Energy Expenditure: Technical Recommendation for Use in Humans, ed Prentice AM (International Atomic Energy Agency, Vienna)* 1990: 193–210.

[42] Weststrate JA (1993). Resting metabolic rate and diet-induced thermogenesis: a methodological reappraisal. Am J Clin Nutr.

[43] De Castro JM (2009). When, how much and what foods are eaten are related to total daily food intake. Br J Nutr.

[44] Troiano RP, Berrigan D, Dodd KW, Masse LC, Tilert T, McDowell M (2008). Physical activity in the United States measured by accelerometer. Med Sci Sports Exerc.

[45] Maraki M, Tsofliou F, Pitsiladis YP, Malkova D, Mutrie N, Higgins S (2005). Acute effects of a single exercise class on appetite, energy intake and mood. Is there a time of day effect?. Appetite.

