# Supplementary material for: The effects of exercise session timing on weight loss and components of energy balance: midwest exercise trial 2
Source: Int J Obes (Lond). 2019 Jul 9;44(1):114–24. doi: 10.1038/s41366-019-0409-x (PMC6925313; doi:10.1038/s41366-019-0409-x)
Supplement: Supplementary file 1 — Supplemental Figure Legend [file 41366_2019_409_MOESM1_ESM.docx]

**Figure S1.** Descriptive results for weight change (%) at months 0, 3.5, 7, and 10 by group, stratified by sex

**Figure S2.** Descriptive results for weight change (%) at months 0, 3.5, 7, and 10 by group, stratified by original study randomization groups.

**Figure S3.** Correlations between weight change and proportions of exercise sessions completed early (A) and late (B).
