# Supplementary figures and images for: The effects of exercise session timing on weight loss and components of energy balance: midwest exercise trial 2
Source: Int J Obes (Lond). 2019 Jul 9;44(1):114–24. doi: 10.1038/s41366-019-0409-x (PMC6925313; doi:10.1038/s41366-019-0409-x)

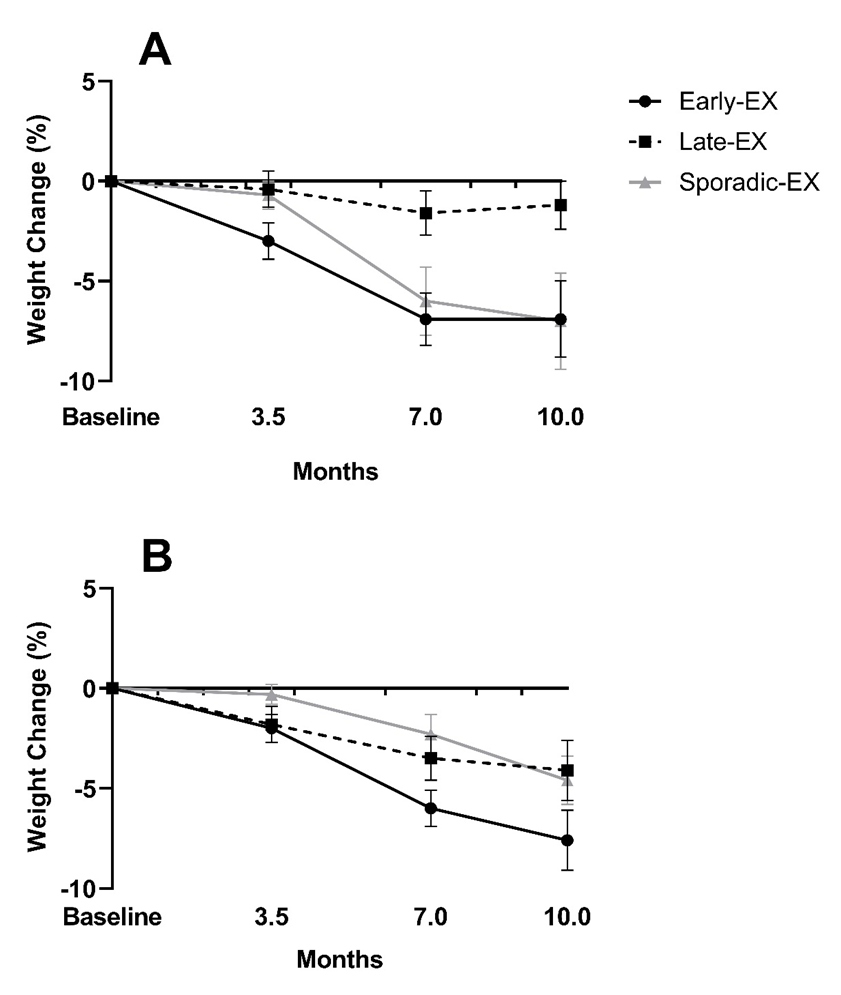

Supplement: Supplementary file 2 — Figure S1 [file 41366_2019_409_MOESM2_ESM.docx]

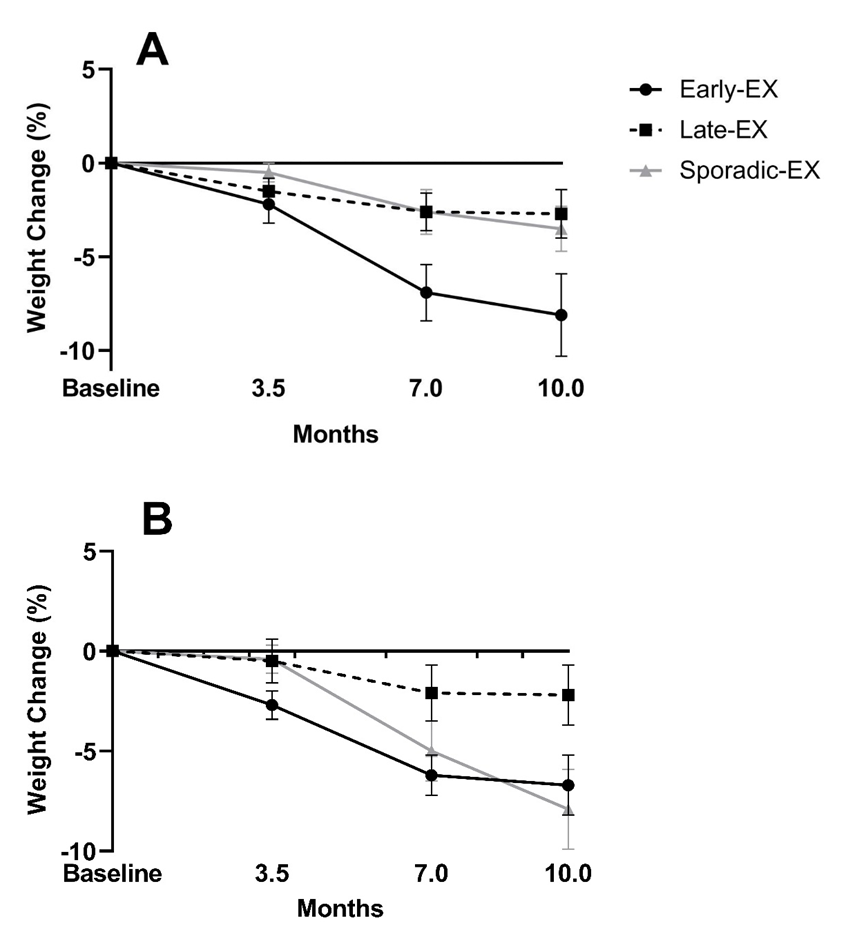

Supplement: Supplementary file 3 — Figure S2 [file 41366_2019_409_MOESM3_ESM.docx]

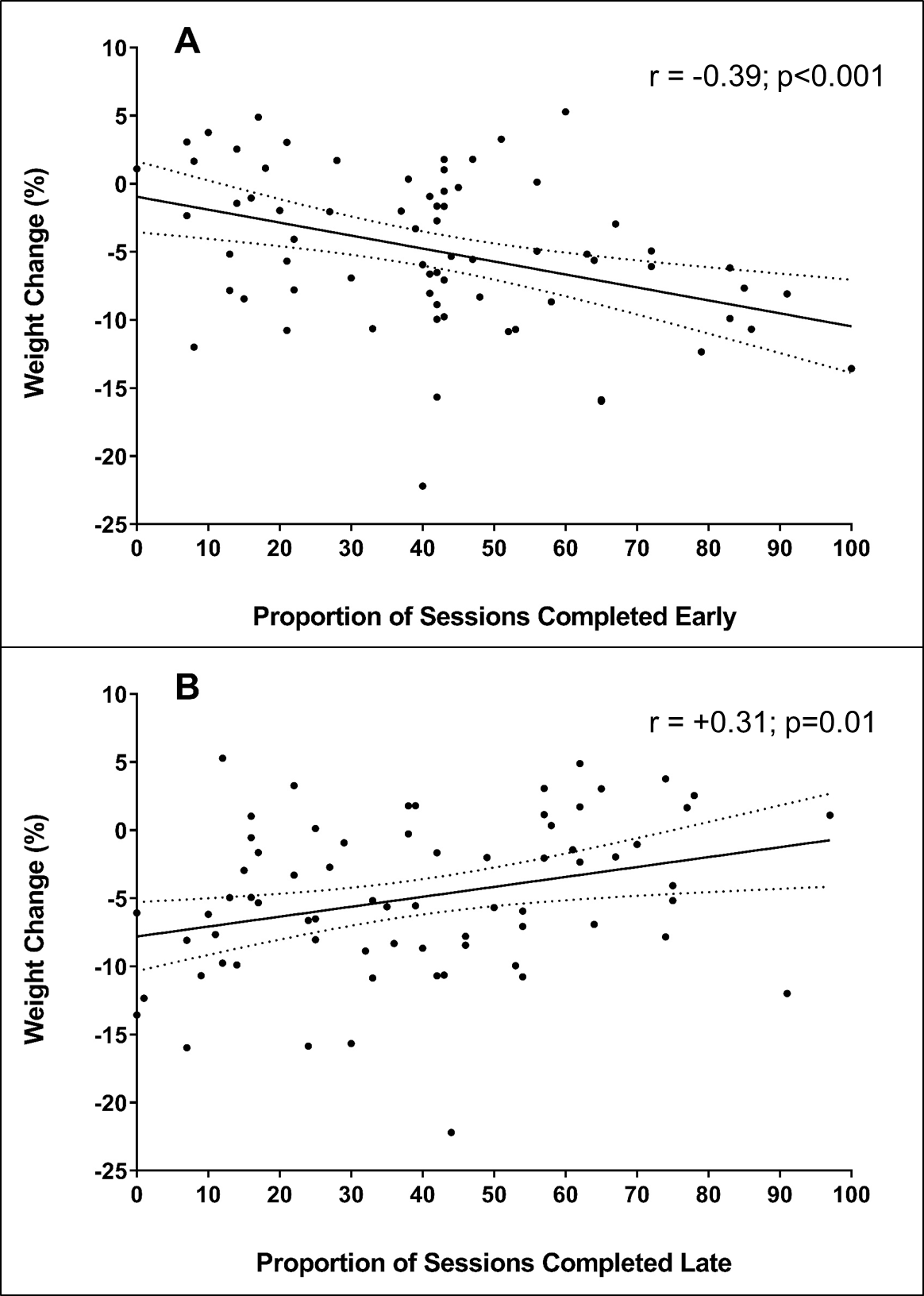

Supplement: Supplementary file 4 — Figure S3 [file 41366_2019_409_MOESM4_ESM.docx]
